# Risk Factors of Hospital-Acquired Pneumonia Among Hospitalized Patients With Cardiac Diseases

**DOI:** 10.7759/cureus.34253

**Published:** 2023-01-26

**Authors:** Mona Alfares, Atheer Almrzouqi, Rana Alghamdi, Raghad Alsharif, Layan Kurdi, Sara Kamfar, Fatmah Alzahrani, Leen Maimani

**Affiliations:** 1 Infectious Diseases, King Abdulaziz University Hospital, Jeddah, SAU; 2 Medicine, King Abdulaziz University Faculty of Medicine, Jeddah, SAU

**Keywords:** cardiac, hospitalized, pneumonia, acquired, hospital, risk

## Abstract

Background

To our knowledge, no studies have been done in Saudi Arabia to determine the risk factors of hospital-acquired pneumonia (HAP) among hospitalized cardiac patients. This study aimed to assess these risk factors.

Methods

A retrospective study was done at King Abdulaziz University Hospital (KAUH), Jeddah, Saudi Arabia. Five hundred hospitalized patients diagnosed with pre-existing cardiovascular disease (CVD) were included. A checklist was used to collect data about patients' demographic characteristics; BMI; smoking and alcohol abuse; type of cardiac disease; other chronic diseases; exposure to immunosuppressives; chemotherapy and radiotherapy in the last six months; glucocorticoid use; application of ventilator; initial, follow-up chest X-ray results; pneumonia vaccination status; nasogastric tube use; general anesthesia received; use of loop diuretics; presence of pulmonary diseases; levels of WBC, erythrocyte sedimentation rate (ESR), and C-reactive protein (CRP); results of blood and respiratory cultures; number of hospitalizations and intensive care unit (ICU) admissions in the last six months; and Richmond Agitation and Sedation Scale (RASS) score.

Results

The prevalence of pneumonia was 7%. Females; patients with autoimmune diseases who were exposed to immunosuppressives or glucocorticoids; those with an initial or second abnormal chest X-ray; patients who used nasogastric tube, had pulmonary disease, and had high levels of WBC, ESR, or CRP; and patients hospitalized for more than two times had a significantly higher percentage of having pneumonia. Abnormal second chest X-ray, high ESR, and more than two times of hospitalization within the last six months were the risk factors of pneumonia on multivariate logistic regression analysis.

Conclusion

Better prevention and intervention programs are needed to assess the risk factors of pneumonia among admitted cardiac patients.

## Introduction

Cardiovascular diseases (CVDs) are chronic diseases that necessitate immediate medical attention when it deteriorates. They include coronary artery disease (CAD), heart failure (HF), cerebrovascular disease, aortic disease (AD), and peripheral vascular disease (PVD) [1,2]. The majority of CVD patients are older and at a high risk of developing hospital-acquired pneumonia (HAP) [3].

Hospital-acquired pneumonia (HAP) is a serious medical condition that manifests as a nosocomial infection 48 hours after admission to the hospital, resulting in increased morbidity and mortality [4-6].

Pneumonia risk was linked to cardiovascular diseases such as chronic heart failure [7]. Mechanical ventilation is the most significant risk factor for developing HAP. Other risk factors for HAP include advanced age, severe underlying illness, long duration of hospital stay, and antibiotic use. HAP is associated with significant mortality. In addition, patients with HAP are subject to increased length of stay in the intensive care unit (ICU) [8].

According to previous studies, HAP incidence is 5.8% among elderly patients in the United Kingdom [9]. The prevalence of HAP was approximately 8% in hospitalized patients with acute heart failure (AHF) [10]. Large-scale cross-sectional survey of nosocomial infections in China showed that the incidence of hospital-acquired infection ranged from 3.22% to 5.22% in hospitalized patients, and the incidence of hospital-acquired lower respiratory tract infection was 1.76%-1.94% [11]. While in other studies, pneumonia-related hospitalization in patients with pre-existing heart failure was 2.72% [12].

Therefore, the presence of HAP in hospitalized patients must be acknowledged. To our knowledge, no study has evaluated HAP risk factors among hospitalized CVD patients in Saudi Arabia.

Hospital-acquired pneumonia is a common respiratory disorder that has a significant impact on the safe recovery of hospitalized patients with cardiovascular disorders. The present study aimed to determine the risk factors of hospital-acquired pneumonia in patients with CVDs.

## Materials and methods

We conducted a retrospective study at King Abdulaziz University Hospital (KAUH), Jeddah, Saudi Arabia, of 500 patients who were admitted from January 2015 to December 2020. The study was approved by the Ethics Committee at King Abdulaziz University (approval number: 484-21).

The inclusion criteria were hospitalized patients above 18 years of age who were admitted to KAUH with a pre-existing cardiovascular disease. The exclusion criteria were patients with no pre-existing cardiovascular disease.

Data was retrieved from the hospital's database (Phoenix) to include patients with cardiac disorders. The included disorders were as follows: all cases of heart failure, non-rheumatic mitral (valve) insufficiency, non-rheumatic mitral (valve) stenosis, aortic (valve) insufficiency or stenosis, acute and subacute infective endocarditis, acute myocardial infarction (MI), angina pectoris, cardiac arrhythmia, atrial fibrillation and flutter, hypertensive heart disease with (congestive) heart failure, chronic ischemic heart disease, supraventricular tachycardia, palpitations, essential (primary) hypertension, cardiac arrest, either hemorrhagic or ischemic stroke, congestive heart failure, atherosclerotic heart disease of the native coronary artery, and ischemic cardiomyopathy.

A pre-designed checklist was prepared to collect data about patients' demographic characteristics (gender, age, and nationality), BMI, smoking and alcohol abuse, type of cardiac disease, chronic diseases, exposure to immunosuppressives, chemotherapy and radiotherapy in the last six months, glucocorticoid use, application of ventilator, initial and second chest X-ray results, and pneumonia vaccine status. Data about nasogastric tube use; receiving general anesthesia; using loop diuretics; the presence of pulmonary diseases; levels of WBC, erythrocyte sedimentation rate (ESR), and C-reactive protein (CRP); results of blood and respiratory cultures; the number of hospitalizations and ICU admissions in the last six months; Richmond Agitation and Sedation Scale (RASS) score (number of sedation days on and off); and the type of pneumonia was also collected.

Data was analyzed statistically using Statistical Package for Social Sciences (SPSS) version 26 (IBM SPSS Statistics, Armonk, NY). To assess the relationship between variables, qualitative data was expressed as numbers and percentages, and the chi-squared (χ^2^) test was used. Quantitative data was expressed as mean and standard deviation (mean ± SD). To assess the risk factors of pneumonia among the studied cardiac patients, multivariate logistic regression analysis was done, and the odds ratio was calculated at a 95% confidence interval (CI). A p-value of 0.05 was considered statistically significant.

## Results

Table 1 shows patients' characteristics; 90.6% were 45 years and above. The incidence of obesity was high (38.6%).

**Table 1:** Distribution of the studied patients according to their demographic characters, BMI, and smoking and alcohol abuse (N = 500) NA: not applicable

Variable	Number (%)
Age	
17-30	8 (1.6)
31-45	39 (7.8)
Above 45	453 (90.6)
Gender	
Female	200 (40)
Male	300 (60)
Nationality	
Non-Saudi	283 (56.6)
Saudi	217 (43.4)
BMI	
18.5 to <25 (healthy weight)	132 (26.4)
25 to <30 (overweight)	168 (33.6)
30 or higher (obese)	193 (38.6)
Less than 18.5 (underweight)	7 (1.4)
Smoking	
Ex-smoker	49 (9.8)
NA	65 (13)
Never	315 (63)
Current smoker	71 (14.2)
Alcohol abuse	
Ex-abuser	3 (0.6)
NA	115 (23)
Never	377 (75.4)
Current abuser	5 (1)

Table 2 shows a comorbidity profile of the study. Heart failure and hypertension were commonly found. Many patients were at risk of having compromised immunity: diabetes mellitus (DM) and previous chemotherapy, radiotherapy, and glucocorticoid therapy.

**Table 2:** Distribution of the studied patients according to the type of cardiac disease, chronic diseases, exposure to immunosuppressives, chemotherapy and radiotherapy in the last six months, glucocorticoid use, the application of ventilator, initial and second chest X-ray results, and pneumonia vaccine status NA, not applicable; MI, myocardial infarction; HTN, hypertension; DM, diabetes mellitus

Variable	Number (%)
Type of cardiac disease	
Heart failure	363 (72.6)
Mitral regurgitation	5 (1)
Mitral stenosis	5 (1)
Aortic regurgitation	1 (0.2)
Aortic stenosis	10 (2)
MI	113 (22.6)
Arrhythmia	70 (14)
Ischemic heart disease	160 (32)
HTN	400 (80)
DM	346 (69.2)
Autoimmune diseases	13 (2.6)
Exposure to immunosuppressives	27 (5.4)
Exposure to chemotherapy within the last six months	14 (2.8)
Exposure to radiation therapy within the last six months	5 (1)
Glucocorticoid use	97 (19.4)
Application of ventilator	
NA	43 (8.6)
No	439 (87.8)
Yes	18 (3.6)
Initial chest X-ray	
Abnormal	100 (20)
NA	222 (44.4)
Normal	178 (35.6)
Second chest X-ray during hospitalization	
Abnormal "consolidation"	63 (12.6)
NA	324 (64.8)
Normal	113 (22.6)
Pneumonia vaccine	
NA	488 (97.6)
No	10 (2)
Yes	2 (0.4)

Table 3 shows ICU procedures: nasogastric tube and general anesthesia; aspiration pneumonia, chronic pulmonary disease, multiple ICU readmissions, and increased length of hospital stay were encountered in patients with HAP.

**Table 3:** Distribution of the studied patients according to nasogastric tube use; receiving general anesthesia; using loop diuretics; the presence of pulmonary diseases; levels of WBC, ESR, and CRP; results of blood and respiratory cultures; the number of hospitalization and ICU admission in the last six months; RASS score (number of sedation days on and off); and the type of pneumonia NA, not applicable; ESR, erythrocyte sedimentation rate; CRP, C-reactive protein; ICU, intensive care unit; RASS, Richmond Agitation and Sedation Scale; AMS, altered mental status; HAP, hospital-acquired pneumonia; VAP, ventilator-associated pneumonia

Variable	Number (%)
Nasogastric tube use	
NA	32 (6.4)
No	452 (90.4)
Yes	16 (3.2)
Receiving general anesthesia	
No	399 (78.8)
Yes	101 (20.2)
Using loop diuretics	
No	131 (26.2)
Yes	369 (73.8)
Pulmonary diseases	
No	375 (75)
Yes	125 (25)
Leukocyte (WBC) count	
High	79 (15.8)
Low	37 (7.4)
Missing	4 (0.8)
Normal	380 (76)
ESR	
High	70 (14)
Low	5 (1)
Missing	324 (64.8)
Normal	101 (20.2)
CRP	
High	204 (40.8)
Low	14 (2.8)
Missing	255 (51)
Normal	27 (5.4)
Blood culture	
NA	307 (61.4)
No organism	193 (38.6)
Respiratory culture	
Endotracheal tube	7 (1.4)
NA	392 (78.4)
Nasopharyngeal swap	10 (2)
Normal	52 (10.4)
Sputum "bacteria"	38 (6.7)
Suction	1 (0.2)
Number of hospitalization within the last six months	
Two times	65 (13)
Less than two times	216 (43.2)
NA	13 (2.6)
More than two times	132 (26.4)
No	74 (14.8)
Number of ICU admission within the last six months	
NA	126 (25.2)
More than nine	1 (0.2)
No	373 (74.6)
RASS score (number of sedation days)	
One-hour AMS, confusion, generalized weakness, and dysarthria	1 (0.2)
NA	415 (83)
No	81 (16.2)
Sedation	3 (0.6)
Number of sedation days off	1.76 ± 2.57
How many days was the patient ventilated	
NA	255 (51)
More than 10	3 (0.6)
No ventilation	242 (48.4)
Type of pneumonia	
Diagnosed with HAP	30 (85.7)
Diagnosed with VAP	1 (2.8)
Diagnosed with aspiration pneumonia	11 (31.4)

**Figure 1:**
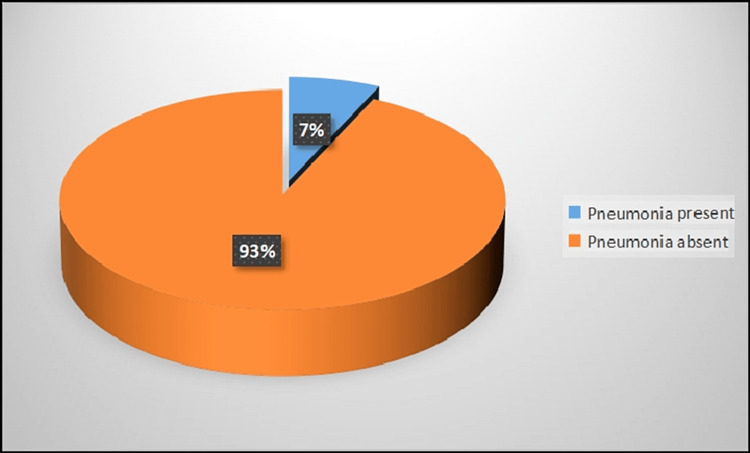
Percentage distribution of pneumonia prevalence among the studied patients

Figure 1 illustrated that the prevalence of pneumonia among the studied cardiac patients was 7%.

Tables 4-5 show that female patients (11.5%) and those having autoimmune diseases (23.1%) and exposure to immunosuppressives (22.2%) and using glucocorticoid (14.4%) had an abnormal initial (15%) or second chest X-ray (25.4%). In addition, patients who used nasogastric tube (50%) had a presence of pulmonary disease (16%) or had high levels of WBC (15.2%), ESR (15.7%), and CRP (11.8%); those whose respiratory culture had bacteria in sputum (28.9%) and who were hospitalized for more than two times (15.9%) had a significant higher risk of hospital-acquired pneumonia (p ≤ 0.05) (Table 6).

**Table 4:** Relationship between the prevalence of pneumonia and patients' demographic characters, BMI, and smoking and alcohol abuse NA, not applicable; χ^2^, chi-squared

Variable	Pneumonia		χ^2^	P-value
	Present number (%)	Absent number (%)		
Age				
17-30	0 (0.0)	8 (100)	1.95	0.376
31-45	1 (2.6)	38 (97.4)		
Above 45	34 (7.5)	419 (92.5)		
Gender				
Female	23 (11.5)	177 (88.5)	10.36	0.001
Male	12 (4)	288 (96)		
Nationality				
Non-Saudi	22 (7.8)	261 (92.2)	0.6	0.439
Saudi	13 (6)	204 (94)		
Smoking				
Ex-smoker	3 (6.1)	46 (93.9)	1.25	0.74
NA	3 (4.6)	62 (95.4)		
Never	25 (7.9)	290 (92.1)		
Current smoker	4 (5.6)	67 (94.4)		
Alcohol abuse				
Ex-abuser	0 (0.0)	3 (100)	1.99	0.573
NA	11 (9.6)	104 (90.4)		
Never	24 (6.4)	353 (93.6)		
Current abuser	0 (0.0)	5 (100)		

**Table 5:** Relationship between the prevalence of pneumonia and the type of cardiac disease, chronic diseases, exposure to immunosuppressives, chemotherapy and radiotherapy in the last six months, glucocorticoid use, the application of ventilator, initial and second chest X-ray results, and pneumonia vaccine status NA, not applicable; MI, myocardial infarction; HTN, hypertension; DM, diabetes mellitus; χ^2^, chi-squared

Variable	Pneumonia		χ^2^	P-value
	Present number (%)	Absent number (%)		
Type of cardiac disease				
Heart failure	29 (8)	334 (92)	1.99	0.158
Mitral regurgitation	1 (20)	4 (80)	1.31	0.252
Mitral stenosis	0 (0.0)	5 (100)	0.38	0.538
Aortic regurgitation	0 (0.0)	1 (100)	0.07	0.784
Aortic stenosis	1 (10)	9 (90)	0.14	0.707
MI	3 (2.7)	110 (97.3)	4.23	0.04
Arrhythmia	6 (8.6)	64 (91.4)	0.3	0.578
Ischemic heart disease	7 (4.4)	153 (95.6)	2.49	0.115
HTN	30 (7.5)	370 (92.5)	0.8	0.668
DM	29 (8.4)	317 (91.6)	3.31	0.19
Autoimmune diseases	3 (23.1)	10 (76.9)	5.29	0.021
Exposure to immunosuppressives	6 (22.2)	21 (77.8)	10.39	0.006
Exposure to chemotherapy within the last six months	3 (21.4)	11 (78.6)	4.6	0.032
Exposure to radiation therapy within the last six months	1 (20)	4 (80)	1.31	0.252
Glucocorticoid use	14 (14.4)	83 (85.6)	10.21	0.001
Application of ventilator	4 (22.2)	14 (77.8)	7.24	0.027
Initial chest X-ray				
Abnormal	15 (15)	85 (85)	12.41	0.002
NA	12 (5.4)	210 (94.6)		
Normal	8 (4.5)	170 (95.5)		
Second chest X-ray during hospitalization				
Abnormal "consolidation"	16 (25.4)	47 (74.6)	38.14	<0.001
NA	16 (4.9)	308 (95.1)		
Normal	3 (2.7)	110 (97.3)		
Pneumonia vaccine	0 (0.0)	2 (100)	0.29	0.865

**Table 6:** Relationship between the prevalence of pneumonia and nasogastric tube use; receiving general anesthesia; using loop diuretics; the presence of pulmonary diseases; levels of WBC, ESR, and CRP; results of blood and respiratory cultures; the number of hospitalization and ICU admission in the last six months; RASS score (number of sedation days on and off); and the type of pneumonia NA, not applicable; χ^2^, chi-squared; ESR, erythrocyte sedimentation rate; CRP, C-reactive protein; ICU, intensive care unit; RASS, Richmond Agitation and Sedation Scale; AMS, altered mental status

Variable	Pneumonia		χ^2^	P-value
	Present number (%)	Absent number (%)		
Nasogastric tube use				
NA	3 (9.4)	29 (90.6)	47.7	<0.001
No	24 (5.3)	428 (94.7)		
Yes	8 (50)	8 (50)		
Receiving general anesthesia	8 (7.9)	93 (92.1)	0.16	0.685
Using loop diuretics	24 (6.5)	345 (93.5)	0.53	0.466
Pulmonary diseases				
No	15 (4)	360 (96)	20.73	<0.001
Yes	20 (16)	105 (84)		
Leukocyte (WBC) count				
High	12 (15.2)	67 (84.8)	11.6	0.009
Low	4 (10.8)	33 (89.2)		
Missing	0 (0.0)	4 (100)		
Normal	19 (5)	361 (95)		
ESR				
High	11 (15.7)	59 (84.3)	9.75	0.021
Low	0 (0.0)	5 (100)		
Missing	18 (5.6)	306 (94.4)		
Normal	6 (5.9)	95 (94.1)		
CRP				
High	24 (11.8)	180 (88.2)	12.89	0.005
Low	0 (0.0)	14 (100)		
Missing	9 (3.5)	246 (96.5)		
Normal	2 (7.4)	25 (92.6)		
Blood culture				
NA	20 (6.5)	287 (93.5)	0.28	0.592
No organism	15 (7.8)	178 (92.2)		
Respiratory culture				
Endotracheal tube	1 (14.3)	6 (85.7)	41.17	<0.001
NA	18 (4.6)	374 (95.4)		
Nasopharyngeal swap	3 (30)	7 (70)		
Normal	2 (3.8)	50 (96.2)		
Sputum "bacteria"	11 (28.9)	27 (71.1)		
Suction	0 (0.0)	1 (100)		
Number of hospitalization within the last six months				
Two times	5 (7.7)	60 (92.3)	24.69	<0.001
Less than two times	5 (2.3)	211 (97.7)		
NA	0 (0.0)	13 (100)		
More than two times	21 (15.9)	111 (84.1)		
No	4 (5.4)	70 (94.6)		
Number of ICU admission within the last six months				
NA	15 (11.9)	111 (88.1)	6.26	0.044
More than nine	0 (0.0)	1 (100)		
No	20 (5.4)	353 (94.6)		
RASS score (number of sedation days)				
One-hour AMS, confusion, generalized weakness, and dysarthria	0 (0.0)	1 (100)	1.48	0.685
NA	27 (6.5)	388 (93.5)		
No	8 (9.9)	73 (90.1)		
Sedation	0 (0.0)	3 (100)		
Number of sedation days off	1 (0.001)	1.79 (2.6)	0.71	0.882
How many days was the patient ventilated				
NA	14 (5.5)	241 (94.5)	2.16	0.339
More than 10	0 (0.0)	3 (100)		
No ventilation	21 (8.7)	221 (91.3)		

Table 7 showed that on doing the multivariate logistic regression analysis to assess the risk factors of pneumonia among the studied patients, having more than two times of hospitalization within the last six months was an independent predictor of pneumonia among the studied cardiac patients.

**Table 7:** Multivariate logistic regression analysis of the risk factors of pneumonia among the studied cardiac patients CI, confidence interval; ESR, erythrocyte sedimentation rate; CRP, C-reactive protein; ICU, intensive care unit

Variable	B	Wald	P-value	Odds ratio (95% CI)
Gender	0.19	0.16	0.686	0.82 (0.32-2.11)
Autoimmune diseases	1.46	1.89	0.169	4.31 (0.53-34.69)
Exposure to immunosuppressives	0.99	0.176	0.184	2.69 (0.62-11.62)
Glucocorticoid use	0.17	0.1	0.752	0.84 (0.29-2.43)
Initial chest X-ray	0.74	1.02	0.312	0.47 (0.11-2.01)
Second chest X-ray during hospitalization: abnormal "consolidation"	2.13	5.71	0.017	8.44 (1.46-48.63)
Nasogastric tube use	3.42	1.09	0.002	30.78 (3.57-65.25)
Pulmonary diseases	1.42	7.08	0.008	4.15 (1.45-11.87)
Leukocyte (WBC) count	1.01	3.52	0.06	0.36 (0.12-1.04)
ESR	1.76	4.92	0.026	0.17 (0.03-0.81)
CRP	0.44	0.18	0.667	0.15 (0.2-3.71)
Respiratory culture	0.02	0.13	0.76	0.1 (0.02-1.1)
Number of hospitalization within the last six months	2.07	10.94	0.001	7.98 (2.33-27.53)
Number of ICU admission within the last six months	0.25	0.26	0.61	1.29 (0.47-3.5)

## Discussion

Previous studies found that the magnitude of risk for pneumonia associated with CVDs was higher in patients aged above 45 years. In addition, the majority of patients are males with comorbidities, such as hypertension, diabetes, and autoimmune disease [13,14].

Life-threatening conditions such as multiple trauma, complicated chronic disease, comorbidities, and state of unconsciousness and also the need for ICU admissions and related treatment methods such as mechanical ventilation and nasogastric tube placement, as well as age and smoking, have been identified as potential risk factors for HAP in several studies [15-17].

The present work revealed that 31.4% of the patients developed aspiration pneumonia after being hospitalized. A prior study looked into ventilator-associated pneumonia (VAP). It was shown that due to the decrease in physiological and immune capabilities, older patients frequently have many comorbidities, which can lead to an increase in hospital length of stay and mechanical ventilation time, increasing the risk of VAP. In patients with mental disorders, the length of stay in the hospital and the time spent on mechanical breathing were much longer. Invasive operations in the ICU have expanded in tandem with the lengthening of hospital stays, increasing patient exposure to the bacterial environment. As a result, the likelihood of VAP has considerably increased. VAP's onset and progression have been aided by the independence and interaction of these elements [18].

Our study showed that patients who were admitted to the ICU and used glucocorticoids, antibiotics, and loop diuretics were at higher risk to develop HAP. The same association between ICU admission and pneumonia was found in a previous study [19].

Diuretic medications may theoretically improve respiratory health outcomes in chronic obstructive pulmonary disease (COPD) through a variety of pathways, but they may also cause respiratory injury by having the ability to raise serum bicarbonate and arterial pH, which can reduce peripheral and central chemoreceptor activity. As a result, hypercapnia is associated with increased risks of respiratory morbidity and mortality in older people according to previous research [20-22].

Age, malnutrition, steroid use, chronic renal failure, anemia, unconsciousness, comorbidity, recent hospitalization, and thoracic surgery are all risk factors for HAP in non-ICU patients [23-25].

In the current study, up to 7% of individuals with CVDs developed pneumonia in which 85.7% of them were diagnosed with HAP; the majority of them were hypertensive (80%). Furthermore, myocardial infarction was found to be significantly related to pneumonia (p = 0.04). On the other hand, heart failure and arrhythmia were discovered to be insignificantly associated with pneumonia. Comparing to a previous case-control study, 36 hospitalized patients with cardiovascular disease and pneumonia were matched with 36 controls who also had cardiovascular disease but did not have pneumonia. Only heart failure was found to be an independent risk factor for pneumonia among all cardiovascular disorders [26].

Patients with HAP were shown to have a history of ICU admission in previous investigations, and multilevel regression analysis revealed that the ICU department was substantially linked with the prevalence of HAP [27,28]. In Western countries, approximately 1.5% of all hospitalized patients developed HAP, while 8.3% of elderly hospitalized patients developed HAP [27].

A limitation of the present study could be the retrospective nature of the study design and the presence of incomplete medical records for some patients.

## Conclusions

Our study evaluated the attributed factors of hospital-acquired pneumonia in cardiovascular disease patients. Pneumonia was found to be present in 7% of individuals with cardiac diseases, and it was shown that HAP was identified as relatively high in hospitalized patients, as well as several associated factors including gender, ICU admission, MI, exposure to immunosuppressives, and glucocorticoid use. Comprehensive, preventive, and intervention strategies are required to assess risk factors for pneumonia in hospitalized cardiac patients.
